# Animal models in tuberculosis metabolomics: a systematic review of current evidence and the road to translational relevance

**DOI:** 10.3389/fmolb.2025.1688882

**Published:** 2025-10-15

**Authors:** Rochelle Caudron, Ilse Du Preez, Laneke Luies, Monique Opperman

**Affiliations:** ^1^ Biomedical and Molecular Metabolism Research, Faculty of Natural and Agricultural Sciences, North-West University, Potchefstroom, South Africa; ^2^ Centre for Human Metabolomics, Desmond Tutu School of Medicine, Faculty of Health Science, North-West University, Potchefstroom, South Africa

**Keywords:** animal models, metabolites, metabolomics, pulmonary tuberculosis, translational potential, TB characterisation, pathway analysis

## Abstract

**Background:**

Animal models are important for tuberculosis (TB) research, offering controlled settings to study disease mechanisms. However, their ability to replicate TB-induced metabolic responses in humans is uncertain. This systematic review evaluated the current use of animal models in metabolomics studies aimed at characterising active pulmonary TB.

**Methods:**

PubMed, Scopus, and Web of Science were systematically searched for metabolomics studies of pulmonary TB in humans and animal models, following PRISMA guidelines. Eligible studies were screened, and quality was assessed using QUDOMICS and STAIR tools. Data were synthesised by species, sample matrix, experimental design, and reported differential metabolites. Differential metabolite names were compared between species and subjected to pathway analysis in MetaboAnalyst 6.0.

**Results:**

Of the 80 eligible studies, nine involved animal models, predominantly mice. These models captured only 4.7% of human TB-associated differential metabolites, with the highest overlap (3.8%) in mouse lung tissue. Despite low concordance at metabolite level, conserved disruptions were observed in amino acid, glutathione, and one-carbon metabolism pathways. Interspecies variation was evident, influenced by host species, sample matrix, infection protocol, and analytical method.

**Conclusion:**

Animal models partially replicated key metabolic features of human TB, particularly at the pathway level. However, variability across studies hampers current translational interpretation. Broader model use, standardised protocols, and integrated multi-platform omics approaches are needed to improve the relevance and comparability of animal models in TB metabolomics research.

## 1 Introduction

Despite ongoing global efforts, pulmonary tuberculosis (TB), caused by *Mycobacterium tuberculosis* (*Mtb*), remains a major health challenge. An improved understanding of TB’s underlying biological mechanisms, and how these vary between individuals, is essential for improving disease detection and deepening our understanding of TB pathogenesis.

“OMICS” approaches (including genomics, proteomics, transcriptomics and metabolomics) have been extensively applied to investigate the complex biological mechanisms of TB, aiming to collectively analyse the structure, function, and interactions of various molecular components in a biological system (Omenn et al., 2012). Metabolomics involves the systematic identification and quantification of small-molecule metabolites within biological matrices such as blood, urine, or tissues, reflecting the body’s current physiological state (Asante-Poku et al., 2024). Due to its strong correlation to the observed phenotype, metabolomics is increasingly employed in disease characterisation, which involves identifying distinct metabolomic patterns that reflect the presence, stage or progression of a disease (Akyol et al., 2023).

However, metabolomics data can be challenging to interpret due to high biological variability. Factors such as age, sex, diet, co-morbidities, microbiome composition, circadian rhythms, and stress can influence metabolite levels (Omenn et al., 2012). For example, it has been reported that older TB patients exhibit distinct metabolic profiles compared to children (Namdeo et al., 2020; Tornheim et al., 2022), while some studies have observed baseline metabolite level differences between males and females infected with TB (Beukes et al., 2023; Carranza et al., 2022). Additionally, inter-individual variations in diet and microbiota composition have been shown to influence short-chain fatty acid and amino acid levels (Du Preez et al., 2017). HIV co-infection has also been shown to significantly alter TB-associated metabolomic signatures (Beukes et al., 2023; Olivier and Luies, 2023). Such variability is often mitigated by using animal models under tightly controlled experimental conditions. By standardising factors like disease severity, environmental influence, genetics, age and nutrition, these models enable more consistent and reproducible investigations (Dube et al., 2020; Singh and Gupta, 2018; Trifonova et al., 2023; Zhan et al., 2017).

Animal models have been widely used in TB research to investigate various aspects of the disease, including pathogenesis, latency, treatment effects and vaccination (Dube et al., 2020; Zhan et al., 2017). However, their application in TB metabolomics is still evolving, with only a limited number of animal model-based studies focusing on the characterisation of TB-induced host metabolome changes (Du Preez et al., 2019). However, metabolome alterations in animal models may differ inherently from those in humans due to interspecies differences and variations in TB pathology. Therefore, it is of particular importance to investigate whether these models can accurately reflect human metabolic responses during *Mtb* infection.

This review aims to summarise and evaluate the use of animal models in TB metabolomics to date, with a particular focus on how well TB-induced metabolite profiles in these models reflect host metabolic changes observed during active pulmonary TB in humans. We explore these studies based on study design, sample types, analytical methods, and the biological relevance of the reported metabolites, ultimately aiming to identify promising approaches and highlight key limitations in the field.

## 2 Methods

A systematic review of pulmonary TB metabolomics studies in human and animal models was conducted in accordance with the PRISMA (Preferred Reporting Items for Systematic Reviews and Meta-Analyses) standards (Page et al., 2021). The completed PRISMA checklist is available in Supplementary Table S1. The systematic review protocol was registered in the International Prospective Register of Systematic Reviews (PROSPERO) under the registration number CRD420251038286. This study was approved by the Research Ethics Committee of the North-West University (NWU-00780-24-A5).

### 2.1 Data sources and search strategy

Three electronic databases—PubMed, Web of Science and Scopus databases—were searched using tailored search strings aligned with each database’s syntax, as outlined in Supplementary Section 1.1. To ensure a comprehensive search, no language or date restrictions were applied during the initial database searches. Reference lists of all publications included in this review, as well as all relevant review articles, were manually screened for additional studies not captured by the primary search. Authors were contacted directly if required data were missing or if study materials were not publicly accessible.

### 2.2 Eligibility criteria

#### 2.2.1 Time period and language

Original research studies published in English up to 02 July 2025 were included.

#### 2.2.2 Study and document type

Eligible study designs included cohort, case-control, and cross-sectional studies, regardless of whether they analysed fresh (prospective) or frozen/biobank (retrospective) samples. Case reports, clinical trials, grey literature, commentaries, letters to the editor, abstracts, and conference proceedings were excluded.

#### 2.2.3 Research model TB type and sample matrix

All studies using metabolomics to investigate metabolome alterations in symptomatic humans and/or animal hosts due to pulmonary infection with any drug-susceptible *Mtb* strains were reviewed. Studies exclusively addressing latent TB infections (LTBI), multidrug-resistant TB (MDR-TB), treatment response, co-morbid conditions, extrapulmonary TB, or vaccine development were excluded. Only *in vivo* biological sample matrices were considered; *in vitro* bacteriological culture studies were excluded.

#### 2.2.4 Ethics approval

Studies were required to demonstrate ethical clearance through institutional approval, documented informed consent, or the use of anonymised or secondary data. Studies lacking appropriate ethical oversight, consent procedures, or those using identifiable data without approval were excluded.

### 2.3 Screening and study selection

Two reviewers independently conducted the database search and research study screening. Database search results were exported in a comma-separated values (CSV) format, which included the bibliographic metadata for each study, and merged into one Microsoft Excel workbook. Duplicate entries were removed based on digital object identifiers (DOIs), with those lacking DOIs assigned temporary numbers and manually checked using title and author metadata. Title and abstract screening were conducted using the stepwise exclusion criteria outlined in Section 2.2. Full-text screening was performed to further assess studies that could not be definitively included or excluded during the title and abstract screening step. Disagreements or uncertainties were resolved through discussion with two additional reviewers.

### 2.4 Data extraction and quality control

#### 2.4.1 Data extraction

A structured Microsoft Form was developed to standardise data extraction across studies. This form captured key features including research model characteristics and experimental approaches (details provided in Supplementary Table S2). Differential metabolites (defined as those showing statistically significant differences between experimental groups as reported by each study) were recorded in an Excel workbook along with the study citation, comparison groups, reference groups, sample types, *Mtb* strain, and direction of regulation.

#### 2.4.2 Metabolite nomenclature harmonisation

To standardise metabolite annotation across studies and enable meaningful comparison of differential metabolites, all reported differential metabolite names were harmonised against the Human Metabolome Database (HMDB, https://hmdb.ca). The aim was to assign a uniform nomenclature using HMDB’s common names where possible.

Initially, all reported metabolite names extracted from the reviewed studies were compared to HMDB entries and their known synonyms using an SQL query in a locally hosted PostgreSQL database. The database included HMDB metabolite names, synonyms, accession numbers, and taxonomic classifications. To support fuzzy matching, the pg_trgm extension was enabled to calculate similarity scores between reported names and HMDB entries.

A similarity search was then performed using a trigram-based matching approach to identify the closest HMDB synonym for each reported metabolite. All matches were manually reviewed to determine whether an appropriate HMDB match could be confidently assigned. Metabolites without acceptable HMDB matches were annotated with the name as reported in the original study.

#### 2.4.3 Study quality and risk of bias assessment

All included studies were subjected to quality and risk of bias assessment using an adapted version of the Quality Assessment of Diagnostic Accuracy Assessment (QUDOMICS) tool (Lumbreras et al., 2008), designed for omics studies (Whiting et al., 2003). Additionally, the Stroke Therapy Academic Industry Roundtable (STAIR) tool (Fisher et al., 2009) was applied to all animal model studies.

The assessment used a quality assurance (QA) scoring system based on 15 criteria for human studies and 22 for animal studies, detailed in Supplementary Table S3. Each criterion was rated as: “Yes” (2 points), “No” (0 points), “Unclear” (1 point), and “Not applicable” (1 point). Human studies scoring ≥25 were classified as high quality, 15–24 as intermediate, and ≤14 as low. Animal studies scoring ≥30 were rated high quality, 18–29 intermediate, and ≤17 low. Only studies with intermediate or high quality were included in the final analysis, as low QA studies pose a risk of reporting unreliable, non-reproducible and biased findings, making it challenging to integrate and compare the findings with those of other studies (Whiting et al., 2003).

### 2.5 Data synthesis and analysis approach

Data were manually extracted and processed using Microsoft 365 platforms (Forms, Excel, and Power BI) and R version 4.2.3. A three-stage analysis strategy was applied.

First, general study characteristics, including cohort composition and animal model details, were summarised. All TB-associated differential metabolites were compiled and stratified by model type and sample matrix. Overlaps between human and animal model metabolites were identified. These comparisons considered metabolites detected across different sample matrices and experimental conditions. It is important to note that the reported overlap percentages depend heavily on the specific differential metabolites captured in each study, which in turn are influenced by the experimental design, analytical platforms, sample types, and study populations.

Second, pathway analysis (PA) was conducted using MetaboAnalyst 6.0 (Pang et al., 2024) for each model and sample matrix. The harmonised compound names of differential metabolites were uploaded to the platform, and targeted pathway analysis was performed. The hypergeometric test was used for pathway enrichment against the MetaboAnalyst reference metabolome, with relevance-betweenness centrality applied for topology evaluation. Species-specific pathway libraries were assigned based on the sample origin: *Homo sapiens* for human and guinea pig data due to key metabolic similarities that are more closely related than to rats or mice (Schyman et al., 2021); *Mus musculus* for mice data; and *Bos indicus* for yellow cattle. Metabolic pathways were deemed significantly altered if both the p-value and FDR were ≤0.05 with an impact value >0.02.

Third, specific metabolite variations were mapped and interpreted for their potential biological and translational relevance in the context of active pulmonary TB.

## 3 Results

### 3.1 Search results

**FIGURE 1:**
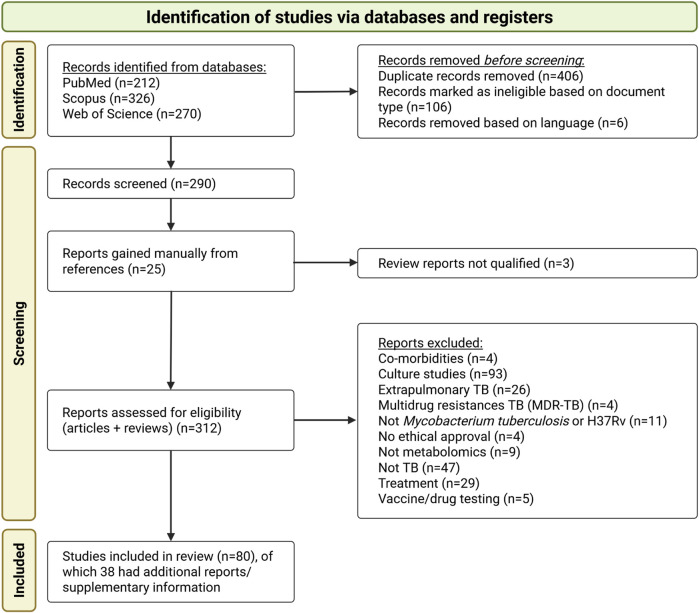
PRISMA flow diagram illustrating the study selection process for the systematic review, outlining the number of publications (studies) and supplementary information (referred to as “reports”) included for full review.

The study selection process is summarised in the PRISMA flowchart (Figure 1). The initial database search identified 808 publications. After removing duplicates and conducting an initial title screening based on the criteria outlined in Section 2.2, 290 publications remained. An additional 22 publications were identified through manual screening of citations from included publications and relevant review publications, resulting in a total of 312 publications for further screening.

The most common reasons for study exclusion were the use of cultured samples, primary focus on drug mechanisms or resistance, investigation of other *Mycobacterium* species, a focus on extrapulmonary TB, or studies evaluating the bioactivity of specific compounds. A total of 80 studies were eligible according to the inclusion and exclusion criteria and were selected for data extraction and synthesis. Supplementary Data from 38 studies were also reviewed to obtain relevant information not reported in the main manuscripts.

### 3.2 Quality assessment results

The quality assessment (Supplementary Table S4) revealed that most studies were of intermediate quality (n = 54), with a smaller number scoring as high-quality (n = 26). Notably, no studies received a low-quality score, indicating that all included studies met the minimum quality criteria required for inclusion in this review.

Cohort sizes varied considerably (Supplementary Table S5). In human studies, participant numbers ranged from 3 to 694, reflecting the heterogeneity of study designs across different clinical and geographical contexts. Animal studies generally had smaller sample sizes, ranging from 6 to 40. Although guidelines recommend at least 10 observations per variable (Memon et al., 2020), sample size requirements in metabolomics research often depend on study context, sample type, and availability. For human studies, 20–30 samples per variable are typically required, although larger cohorts are preferred for clinical studies to enable robust biomarker discovery (Rakusanova and Cajka, 2024). However, biological and non-biological variability, costs, and recruitment challenges frequently limit cohort size. In contrast, animal studies benefit from a controlled environment, making it feasible to use smaller sample sizes (5–10 samples per variable) while balancing ethical considerations (Rakusanova and Cajka, 2024).

A significant gap in the animal studies was the lack of transparency regarding sample size determination. None of the animal studies reported how their sample sizes were calculated, nor did they state whether any animals were excluded from analysis. Only one animal study provided explicit inclusion and exclusion criteria (Fernández-García et al., 2020). The lack of power calculations raises concerns regarding the risk of underpowered analyses and the potential for both Type I and Type II errors (Memon et al., 2020).

### 3.3 Data generated from the search

#### 3.3.1 General study characteristics

**FIGURE 2:**
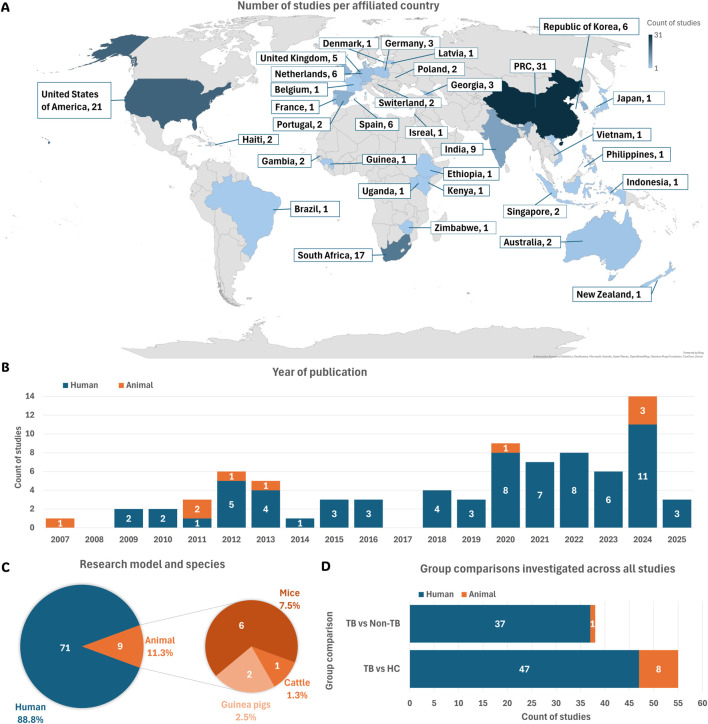
Summary of study characteristics. **(A)** Geographic distribution of studies based on author affiliations. **(B)** Publications per year, categorised by human and animal model studies. **(C)** Total number of studies using human participants or animal models, along with species used across all animal studies. **(D)** Types of group comparisons investigated in each eligible study. Abbreviations: HC, healthy controls; non-TB, non-tuberculosis controls; PRC, People’s Republic of China; TB, tuberculosis.

Based on the affiliation information of all authors listed in each study, most study contributions were made by researchers from the People’s Republic of China (PRC), followed by the United States of America (USA), South Africa, and India (Figure 2A). Country assignment was not limited to the first or corresponding author but included all listed affiliations per study. The earliest eligible publications included in this review were published in 2007 in the USA (Jain et al., 2007) (Figure 2B). From 2009 onward, the number of studies steadily increased.

This systematic review included 71 studies (88.8%) involving human participants and nine studies (11.3%) using animal models (Figure 2C).

The metabolome comparisons between TB-positive and TB-negative groups in each study were classified into two categories: TB versus healthy controls (HC) and TB versus non-TB groups (Figure 2D). Table 1 summarises the group definitions used throughout the review.

**TABLE 1:** Group definitions of human and animal studies investigating tuberculosis.

Abbreviation	Full term	Description of participants or experimental models
TB-positive		
*Mtb* infection		
TB	Tuberculosis	Active, drug-susceptible pulmonary TB caused by *Mtb* infection
TB_Dxx TB_Wxx	TB (duration specified)	Active *Mtb* infection assessed at defined time points post-experimental infection. Time points are denoted by D (days) or W (weeks); e.g., TB_D15 refers to 15 days, and TB_W4 to 4 weeks after infection
TB-negative (controls)		
Healthy controls		
HC	Healthy controls	No history of *Mtb* infection or other major health conditions. Animal models were not infected
HHC	Healthy household contacts	Close contacts of active TB patients without evidence of *Mtb* infection or active disease
Non-TB controls		
Non-TB	Non-tuberculosis infection	Displaying TB-like symptoms but negative diagnostic test results for *Mtb* infection; also include unconfirmed LTBI, NTM, and other respiratory diseases
NTM	Non-tuberculous mycobacterial	Infected with a mycobacterial strain other than *Mtb*
LTBI	Latent TB infection	Confirmed infection with *Mtb* without clinical signs of active disease

Abbreviations: *Mtb*, *Mycobacterium tuberculosis*; NTM, non-tuberculous mycobacterial TB, tuberculosis.

#### 3.3.2 Research model characteristics

##### 3.3.2.1 Human participants used in TB metabolomics

**FIGURE 3:**
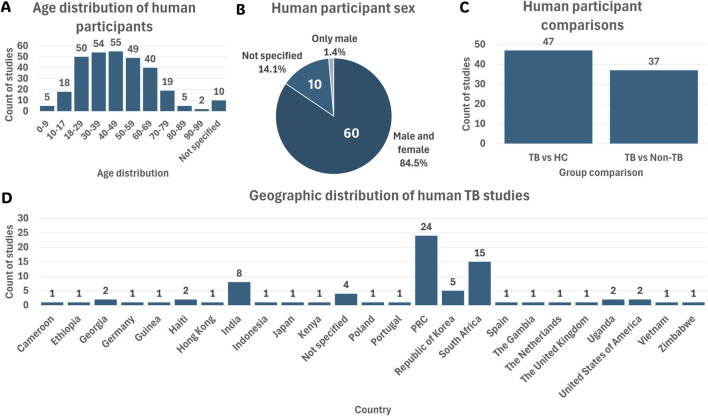
Summary of the 71 human cohort study characteristics. **(A)** Age distribution of participants across all human studies. **(B)** Sex distribution of human participants. **(C)** Group comparisons investigated in human studies. **(D)** Country-wise population distribution of human TB studies. Abbreviations: HC, healthy controls; *Mtb*, *Mycobacterium tuberculosis*; non-TB, non-tuberculosis; PRC, People’s Republic of China; TB, tuberculosis.

Among the eligible human studies, the predominant focus was on participants aged 18–59 years (Figure 3A). This aligns with the global burden of TB, as this age group is most affected by the disease (Yang et al., 2024). Furthermore, most studies (84.5%) included both males and females (Figure 3B). A diverse range of populations was investigated across the reviewed studies, with the majority encompassing participants from PRC (33.8%) and South Africa (21.1%) (Figure 3D). This population distribution aligns closely with the locations of the associated research sites mentioned in Section 3.3.1, highlighting regional focuses in TB metabolomics research.

##### 3.3.2.2 Animal models used in TB metabolomics

**FIGURE 4:**
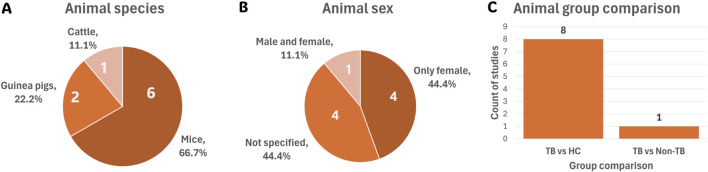
Summary of the nine animal model studies’ cohort characteristics. **(A)** Species used across all animal studies. **(B)** Sex distribution of animals used. **(C)** Group comparisons investigated in animal studies. Abbreviations: HC, healthy controls; TB, tuberculosis.

Among the nine eligible animal studies, mice were the predominant species used (66.7%), followed by guinea pigs (22.2%) and cattle (11.1%) (Figure 4A). Regarding sex distribution, 44.4% of the animal studies used female animals, 44.4% did not specify sex, and only 11.1% included both male and female animals (Figure 4B). The majority of animal studies investigated TB versus HC (88.9%), followed by TB versus non-TB (11.1%) comparisons (Figure 4C).

###### 3.3.2.2.1 Mouse models

In this review, the C57BL/6 strain was the most used mouse strain, appearing in three studies (33.3%) (Table 2). Other strains, including humanised NSG-SGM3, BALB/c, and specific pathogen-free (SPF) C57BL/6, were each used in only one study (11.1%).

**TABLE 2:** Summary of Mtb infection characteristics used in animal studies.

Animal species strain	*Mtb* infection				Study citation
	Strain	Dose	Route	Duration	
Cattle					
Yellow	H37Rv	Very high (>10,000 CFU)	Intratracheal	33 weeks	Chen et al. (2013)
Guinea pigs					
Out-bred Hartley	H37Rv	Very high (>10,000 CFU)	Aerosol	15, 30 and 60 days	Somashekar et al. (2011)
SPF Albino Hartley	W-Beijing strain	Low (20–120 CFU)	Aerosol	3 weeks	Somashekar et al. (2012)
Mice					
BALB/c	Erdman strain	High (100–10,000 CFU)	Aerosol	19 days	Jain et al. (2007)
C57BL/6	H37Rv	Low (20–120 CFU)	Aerosol	3 weeks	Chaudhary et al. (2024)
C57BL/6	H37Rv	Very high (>10,000 CFU)	Tail vein	10 weeks	Zhang et al. (2024b)
C57BL/6	H37Rv	Very high (>10,000 CFU)	Intratracheal	4 and 9 weeks	Fernández-García et al. (2020)
Humanised NSG-SGM3	H37Rv	Low (20–120 CFU)	Aerosol	15 days; 4 and 5 weeks	Bohórquez et al. (2024)
SPF C57BL/6	H37Rv	Moderate (120–1,000 CFU)	Aerosol	4 weeks	Shin et al. (2011)

CFU, colony forming unit; *Mtb*, *Mycobacterium tuberculosis*; SPF, specific pathogen free.

C57BL/6 and BALB/c mice are among the most used strains in TB research. While these strains exhibit distinct immune responses to *Mtb* infection, both models typically develop inflammatory, non-necrotic pulmonary lesions in which bacilli are primarily contained within host cells. This differs from human TB disease and certain other animal models, where necrotic granulomas form and bacilli predominantly accumulate extracellularly within these structures (Singh and Gupta, 2018).

Furthermore, C57BL/6 mice tend to be more resistant to *Mtb*, showing prolonged survival and reduced bacterial burden after the onset of adaptive immunity (Li and Li, 2023; Singh and Gupta, 2018). In contrast, BALB/c mice are more susceptible and thus useful for studying disease progression and immune responses. However, their limited capacity to model latency poses a challenge, as infection typically results in mild local inflammation, strong systemic immune activation, poor bacterial control, and progressive lung damage (Li and Li, 2023; Zhang et al., 2023).

Humanised NSG-SGM3 mice, which express human cytokines, support full immune cell differentiation and exhibit immune responses more similar to humans, making them particularly valuable for studying human-specific immune responses and evaluating human-targeted therapies (Bohórquez et al., 2024; Li and Li, 2023).

Interestingly, two of the C57BL/6 mouse studies (Chaudhary et al., 2024; Zhang et al., 2024b) exclusively used female mice, while one study (Fernández-García et al., 2020) included both male and female mice (Figure 4B). Notably, sex-based differences in TB progression have been observed in mice, highlighting the importance of reporting and considering sex as a biological variable in study design. For example, Bini et al. (2014) found that male C57BL/6 mice were more susceptible to *Mtb* H37Rv infection via the intratracheal route than females. The study suggested that testosterone may modulate immune responses, as castration reduced this increased susceptibility. Supporting these findings, Dibbern et al. (2017) reported that male mice experienced more rapid disease progression, higher bacterial loads, and increased morbidity and mortality. This was associated with an early and exaggerated pulmonary inflammatory response, resulting in more severe pathology.

Additionally, one study used an SPF female mouse model (Shin et al., 2011) (Table 2). SPF models are raised in controlled environments free from known pathogens, reducing confounding factors such as natural infections, microbiota variability, and baseline immune activation. This ensures that the immune and metabolic responses observed in experiments are primarily due to the disease under investigation and the experimental interventions applied (Huggins et al., 2019).

###### 3.3.2.2.2 Guinea pigs

Guinea pigs were used in only two TB metabolomics studies to date (Figure 4A), both conducted by the same research group (Somashekar et al., 2011; Somashekar et al., 2012) (Table 2), highlighting their marked underutilisation. This limited use may stem from practical challenges in housing and maintaining guinea pigs compared to murine models. Guinea pigs require larger enclosures, a specialised diet, and tighter environmental controls—particularly in terms of temperature and humidity—to ensure their health and welfare (Weichbrod et al., 2018). Only one of the two studies reported the sex, indicating that female guinea pigs were used. The study did not provide a rationale for using females, and no direct link was found in the literature. This omission limits interpretation, as sex-related physiological differences may influence immune and metabolic responses.

The two studies used different guinea pig strains: SPF Albino Hartley and outbred Hartley guinea pigs (Table 2). SPF Albino Hartley guinea pigs are inbred, pathogen-free, and genetically uniform, making them suitable for experiments requiring strict control over biological variability. In contrast, outbred Hartley guinea pigs offer greater genetic diversity and are not bred under pathogen-free conditions, potentially better reflecting the biological heterogeneity seen in human populations (Lan et al., 2020).

###### 3.3.2.2.3 Cattle

One reviewed study used cattle (unspecified sex) as an animal model in TB metabolomics research (Chen et al., 2013) (Figure 4A). Cattle are natural hosts for *Mycobacterium bovis* (*M. bovis*) and offer unique advantages for studying TB pathogenesis and evaluating therapeutic interventions (Li and Li, 2023). In the study by Chen et al. (2013) (Table 2), cattle were experimentally infected with *Mtb* H37Rv, a clinical *Mtb* strain, and *M. bovis* to investigate differences in virulence among these *Mtb* strains.

##### 3.3.2.3 Mtb infection characteristics

In animal models, infection-related variables—including *Mtb* strain, dose, route of administration, and infection duration—significantly influence disease severity, immune responses, and, in turn, the host metabolome. These characteristics are summarised in Table 2.

The laboratory strain H37Rv, isolated in 1905 (Koch, 1884), was used in seven of the nine reviewed studies (Table 2). While phenotypically similar to the original *Mtb* strain (Koch, 1884), H37Rv differs from many clinical strains, which typically grow faster and produce greater quantities of the Early Secreted Antigenic Target 6 kDa (ESAT-6) virulence factor, known to modulate both host immunity and metabolism (Chiner-Oms et al., 2018).

The Erdman strain, also used in one reviewed study (Table 2), is characterised by consistent virulence in mice and robust granuloma formation, making it suitable for vaccine and pathogenesis research (Via et al., 2013). The W-Beijing strain, associated with MDR-TB and hypervirulence, is particularly valuable in studies of drug resistance, transmission, and immune evasion (Hanekom et al., 2011).

Infection in animal models is typically induced via aerosol, intranasal, or intratracheal routes to replicate natural human transmission (Li and Li, 2023). Among the reviewed animal model studies, aerosol infection was most common (66.7%), followed by intratracheal (22.2%) and tail vein (11.1%) administration (Table 2).

Both the infection dose and route can significantly impact disease pathology and host immune responses (Li and Li, 2023). For instance, intravenous infection in mice often results in higher bacterial loads and more severe pathology compared to aerosol infection (Nikonenko et al., 2004). C57BL/6J mice infected via aerosol typically exhibit contained infection, characterised by necrotic lesions, low bacterial loads, and limited inflammation (Li and Li, 2023). In contrast, BALB/c mice tend to require higher intratracheal infectious doses and develop more severe pathology, including progressive lung consolidation, fibrosis, elevated T-cell infiltration, and increased anti-inflammatory cytokine expression (Li and Li, 2023). This aligns with the reviewed studies involving C57BL/6 and humanised NSG-SGM3 mice. Aerosol doses ranged from low (20–120 CFU) to moderate (120–1,000 CFU), while very high doses (>10,000 CFU) were administered via intratracheal or tail vein routes (Table 2). Controversially, the BALB/c model received a high infectious dose even via the aerosol route.

In guinea pigs, aerosol infection leads to granulomas that closely resemble human TB histopathology (Li and Li, 2023). Disease progression in this model is also dose-dependent. Somashekar et al. (2012) used both a low infection dose to investigate metabolic signatures for non-invasive diagnostic or prognostic purposes, as well as a very high dose to characterise broader metabolomic alterations (Somashekar et al., 2011) (Table 2). Low-dose infections tend to result in chronic, slowly progressing disease, whereas high-dose exposures accelerate progression and increase mortality risk (Li and Li, 2023). However, high-dose models may not accurately reflect natural transmission, potentially limiting their translational relevance.

In cattle, infection is typically induced using very high-dose aerosol (as is the case in the included cattle study, Table 2) or intratracheal administration, compensating for the low natural shedding of *M. bovis*. Lesions primarily affect the lungs and lymph nodes and are often caseous and mineralised. As in other models, lesion distribution and severity are influenced by both route and dose (Li and Li, 2023).

#### 3.3.3 Metabolomics study flow

**FIGURE 5:**
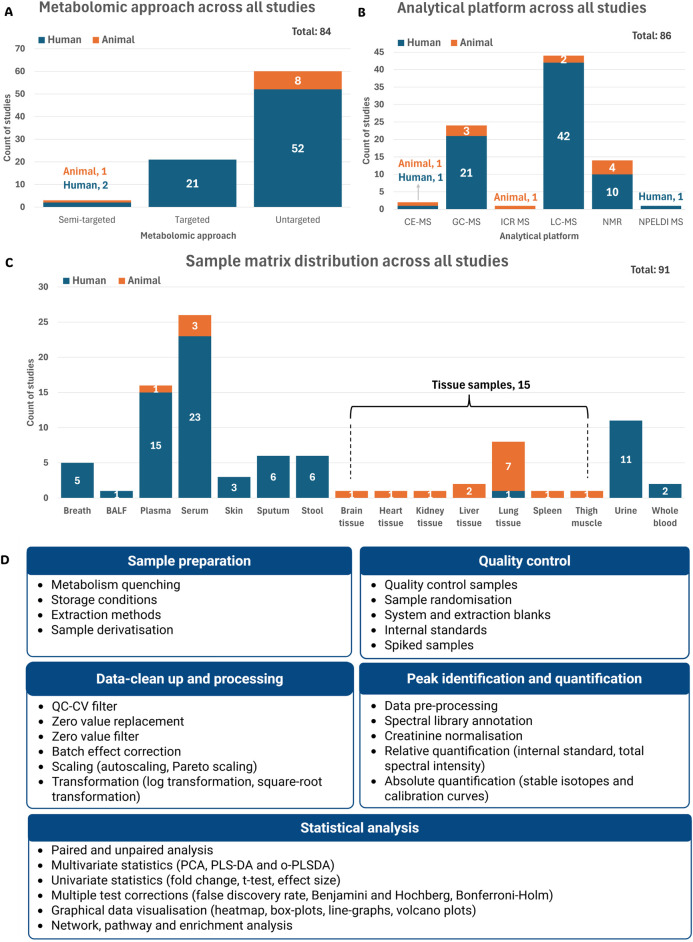
Summary of the metabolomics workflows used in the reviewed studies. The total represents the number of recorded parameters within each category as reported across all 80 reviewed studies. **(A)** Distribution of metabolomics approaches across all studies. **(B)** Analytical platforms used in the reviewed studies. **(C)** Sample matrices used for metabolomics analysis across all studies. **(D)** Summary of sample and data analysis variables used mostly in the reviewed studies (detail given in supplementary information) Abbreviations: BALF, bronchoalveolar lavage fluid; CE-MS, capillary electrophoresis mass spectrometry; GC-MS, gas chromatography mass spectrometry; ICR MS, ion cyclotron resonance mass spectrometry; LC-MS, liquid chromatography mass spectrometry; NMR, nuclear magnetic resonance; NPELDI MS, nanoparticle-enhanced laser desorption/ionisation mass spectrometry; QC-CV, quality control coefficient of variation; PCA, principal component analysis; PLS-DA, partial least squares discriminant analysis; o-PLSDA, orthogonal partial least squares discriminant analysis.

The choice of metabolomics approach, analytical platform, sample matrix and data analysis method can significantly impact the metabolomics signatures detected during *Mtb* infection. To illustrate the range of methods used, general workflows from the reviewed studies are summarised in Figures 5A–D, while Supplementary Table S5 details the specific characteristics of each study.

Some studies reported multiple experimental parameters, for example, using more than one metabolomics approach, sample matrix, or analytical platform (Supplementary Table S5). Each unique instance was recorded separately, resulting in a total number of entries exceeding the number of reviewed studies (n = 80). Accordingly, the percentages reported in this section represent the distribution of recorded parameters within each category, rather than the total number of studies.

Cohort size was also documented for each study, given its influence on statistical power, reliability, and reproducibility (Supplementary Table S5). A small sample size can increase the likelihood of false positives or negatives, while overly large studies without adequate design can lead to unnecessary cost and data complexity (Dunn et al., 2015).

##### 3.3.3.1 Metabolomics approach

Metabolomics studies usually follow untargeted, targeted, or semi-targeted approaches. Here, most reviewed cases (71.4%) used an untargeted approach, suggesting a strong emphasis on exploratory, hypothesis-generating research (Figure 5A). This method captures a broad spectrum of metabolites without prior selection, offering the potential to uncover novel biomarkers relevant to TB diagnosis and disease characterisation (Du Preez et al., 2019; Roach et al., 2024). In contrast, targeted metabolomics—used in 25.0% of cases (all human) — focuses on the precise quantification of predefined metabolites (Figure 5A). Despite its narrower scope, this approach improves sensitivity and specificity through the optimisation and enhancement of specific preparation and analysis methods (Roach et al., 2024).

Only 3.6% of cases used a semi-targeted approach (Figure 5A), combining features from both untargeted and targeted strategies. This approach enables the identification and quantification of groups of metabolites within specific pathways, without the prior knowledge of the exact metabolites of interest (Billet et al., 2020; Roach et al., 2024).

##### 3.3.3.2 Analytical platforms

High-resolution analytical platforms such as mass spectrometry (MS), often coupled with various separation systems, and nuclear magnetic resonance (NMR), remain the most widely used in metabolomics (Wishart et al., 2022). In this review, liquid chromatography coupled to MS (LC-MS) was the predominantly used analytical platform (51.2%), followed by gas chromatography-mass spectrometry (GC-MS, 27.9%) and NMR (16.3%) (Figure 5B).

MS, specifically when coupled with chromatography, offers high sensitivity and specificity, making it ideal for targeted analyses (Munjal et al., 2022). However, it requires extensive sample preparation and is destructive to biological samples. In contrast, NMR is non-destructive, requires minimal preparation, and is highly reproducible, though it has lower sensitivity and limited resolution in complex samples like those encountered in TB studies (Munjal et al., 2022).

Less commonly used platforms included capillary electrophoresis mass spectrometry (CE-MS), ion cyclotron resonance mass spectrometry (ICR-MS), and nanoparticle-enhanced laser desorption/ionisation MS (NPELDI-MS), showing the vast number of analytical platforms available in metabolomics (Figure 5B).

##### 3.3.3.3 Sample matrices

Biological sample matrices used in metabolomics range from non-invasive or minimally invasive types such as breath, stool, and urine, to more invasive ones like sputum, blood, and tissue samples (Smith et al., 2020). Each matrix provides distinct metabolic profiles influenced by physiological function and excretion mechanisms.

Blood is the most frequently used matrix in TB metabolomics studies due to standardised collection methods and its ability to reflect systemic changes during infection and treatment (Du Preez et al., 2019). Among the reviewed studies, including human and animal model studies, serum was used in 26 cases (28.6%), plasma in 16 (17.6%) and whole blood in only two (2.2%) (Figure 5C).

Urine, used in 11 cases (12.1%) (Figure 5C), is a non-invasive, readily available matrix that requires minimal sample preparation and reflects host metabolism, although it contains few mycobacterial metabolites. Urine samples, however, present challenges with metabolite normalisation due to natural variability in individual excretion rates (Du Preez et al., 2019), necessitating correction of metabolite levels to creatinine concentration (Nam et al., 2020).

Stool, used in just 6 cases (6.6%) (Figure 5C), is still considered an emerging matrix in TB research, particularly for investigating gut-lung immune interactions and potential diagnostic markers (Luo et al., 2023).

Although sputum samples offer direct access to the infection site, providing valuable metabolomics information about both the bacteria and the host, it was used in only 6 cases (6.6%) (Figure 5C). This is likely due to challenges in the collection—especially in children and immunocompromised patients—and the high viscosity of sputum, which complicates sample processing (Du Preez et al., 2019).

Tissue samples, used in 15 cases (16.5%), offer insights into localised metabolic changes at the infection site (Figure 5C). However, collection is highly invasive and, for lung tissue, limited to pulmonary TB research (Du Preez et al., 2019). Expectedly, most tissue samples were lung tissue (53.3%), with only one human study included (Figure 5C). Alternatively, pleural and bronchoalveolar lavage fluid (BALF) are considered less invasive alternatives for assessing localised pathological processes in human participants. BALF, for example, provides access to the alveolar lining fluid without the need for a pleural puncture (Tokar et al., 2017).

Breath samples, used in 5 cases (5.5%) (Figure 5C), represent a non-invasive matrix ideal for detecting volatile compounds, including in vulnerable populations. However, their use is limited by dilute metabolite concentrations and the lack of standardised sampling and processing procedures (Du Preez et al., 2019). Collection from animal models remains especially challenging, thus limiting use to human studies. Interestingly, skin was more recently used (3.3%) (Figure 5C) to identify metabolic biomarkers differentiating TB patients from HC, demonstrating its potential use in future research (Makhubela et al., 2023; Vishinkin et al., 2021; Wooding et al., 2025), albeit this sample matrix’s use in TB animal model metabolomic studies is not yet clear.

##### 3.3.3.4 Sample preparation, quality control and data analysis variables

Depending on the specific metabolomics approach, analytical platform, and sample matrix, different strategies are followed to identify and analyse the metabolomes of interest. These strategies include procedures for sample preparation, quality control during analysis, and peak identification and quantification. Additionally, the study aims and cohort design influence the selection of data-cleaning methods, statistical analyses, and threshold cut-offs applied to identify differential metabolites. Figure 5D provides an overview of the most frequently used methods across these workflow steps, as identified in the reviewed studies. A detailed summary of study-specific methodologies is presented in Supplementary Table S5.

#### 3.3.4 TB-induced differential metabolites

A total of 7,770 differential metabolite entries associated with TB characterisation were captured across all reviewed studies. Of these, 6924 metabolite entries originated from human studies, representing 3657 distinct metabolites after harmonising metabolite nomenclature (detailed per sample matrix in Supplementary Table S6). Animal model studies contributed 828 metabolite entries, corresponding to 417 distinct metabolites following nomenclature harmonisation (detailed per sample matrix in Supplementary Table S7).

A comparison between human and animal model studies revealed limited overlap in reported differential metabolites. Of the 3657 distinct TB-associated metabolites identified in human studies (across all sample matrices), only 172 (4.7%) were also reported in animal models (Supplementary Table S7). This overlap varied by species. Mouse models demonstrated the highest degree of concordance, with 3.8% of human metabolites also identified in mouse lung tissue (Supplementary Table S7). In contrast, guinea pig and cattle models showed minimal overlap, which may reflect species-specific metabolic responses or the limited number of available studies for these models.

##### 3.3.4.1 Pathway analysis

To assess the specific host metabolic alterations induced by *Mtb*, pathway analysis (PA) was performed on the harmonised differential metabolite annotated list in TB versus control groups across multiple biological matrices. Breath and various mouse tissues (including heart and thigh) were excluded from PA due to insufficient metabolite coverage, while meaningful enrichment results were obtained for matrices such as blood, urine, BALF, stool, and various animal tissues analysed (lung, liver and spleen) (Table 3). A comprehensive table with statistical significance is depicted in Supplementary Table S8. Although sample matrices, including skin, sputum, and mouse blood, kidney and brain tissue, were included in the PA, they did not reveal any significantly altered pathways and are thus not shown here.

**TABLE 3:** Significantly enriched metabolic pathways based on pathway analysis of differential metabolites comparing TB-positive and TB-negative (control) groups across multiple biological matrices. Values indicate the number of differential metabolites detected relative to the total number of known compounds in each pathway.

Significantly affected pathways	Percentage of differential metabolites identified within each pathway (n = total metabolites in the pathway)			
	Human	Cattle	Guinea pig	Mice
Alanine, aspartate and glutamate metabolism	n = 28			
BALF	39.3%	-	-	-
Blood*	35.7%	-	14.3%	-
Lung tissue	-	-	10.7%	35.7%
Spleen tissue	-	-	-	21.4%
Stool	35.7%	-	-	-
Urine	28.6%	-	-	-
Arginine and proline metabolism	n = 36			
BALF	36.1%	-	-	-
Blood*	27.7%	-	-	-
Lung tissue	-	-	-	25%
Arginine biosynthesis	n = 14			
BALF	64.3%	-	-	-
Blood*	71.4%	-	21.4%	-
Lung tissue	-	-	-	50%
Spleen tissue	-	-	-	28.6%
Stool	50%	-	-	-
Urine	42.9%	-	-	-
β-Alanine metabolism	n = 21			
BALF	38.1%	-	-	-
Lung tissue	-	-	-	28.6%
Urine	33.3%	-	-	-
Cysteine and methionine metabolism	n = 33			
Urine	21.2%	-	-	-
Galactose metabolism	n = 27			
BALF	29.6%	-	-	-
Blood*	33.3%	-	-	-
Glutathione metabolism	n = 28			
BALF	32.1%	-	-	-
Blood*	32.1%	-	-	-
Lung tissue	-	-	14.3%	32.1%
Glycerophospholipid metabolism	n = 36			
Blood*	27.8%	-	-	-
Lung tissue	-	-	-	27.8%
Glycine, serine and threonine metabolism	n = 33	n = 34	n = 33	n = 34
BALF	30.3%			
Blood*	36.4%	8.8%	-	-
Lung tissue	-	-	12.1%	26.5%
Urine	27.3%	-	-	-
Glyoxylate and dicarboxylate metabolism	n = 32			
BALF	25%	-	-	-
Blood*	25%	9.4%	-	-
Lung tissue	-	-	-	28.1%
Urine	21.9%	-	-	-
Histidine metabolism	n = 16			
Lung tissue	-	-	-	31.3%
Stool	50%	-	-	-
Urine	43.8%	-	-	-
Linoleic acid metabolism	n = 5			
Blood*	80%	-	-	-
Nicotinate and nicotinamide metabolism	n = 15			
Blood*	40%			
Liver tissue	-	-	-	20%
Spleen tissue	-	-	-	20%
Stool	46.7%	-	-	-
Urine	40%	-	-	-
One carbon pool by folate	n = 26			
BALF	30.8%	-	-	-
Blood*	30.8%	11.5%	-	-
Lung tissue	-	-	11.5%	26.9%
Stool	38.5%	-	-	-
Urine	26.9%	-	-	-
Pantothenate and CoA biosynthesis	n = 20			
BALF	35%			
Urine	35%	-	-	-
Phenylalanine, tyrosine and tryptophan biosynthesis	n = 4			
Liver tissue	-	-	-	50%
Spleen tissue	-	-	-	50%
Purine metabolism	n = 70			
BALF	28.6%	-	-	-
Stool	24.3%	-	-	-
Pyrimidine metabolism	n = 39			
BALF	30.8%	-	-	-
Pyruvate metabolism	n = 23			
Blood*	-	-	13%	-
Lung tissue	-	-	-	30.4%
Sphingolipid metabolism	n = 32			
Blood*	31.3%	-	-	-
Tricarboxylic acid (TCA) cycle	n = 20			
BALF	30%	-	-	-
Lung tissue	-	-	-	30%
Liver tissue	-	-	-	15%
Spleen tissue	-	-	-	15%
Urine	30%	-	-	-
Valine, leucine and isoleucine degradation	n = 40			
Liver tissue	-	-	-	10%
Spleen tissue	-	-	-	10%

Blood * includes whole blood, serum, and plasma. “-” indicates no data available, not analysed, or no significant enrichment. “n” indicates the total metabolites assigned to the pathway (species-specific pathway size according to MetaboAnalyst). Total compounds in pathways may differ between species due to species-specific pathway coverage in MetaboAnalyst. Abbreviations: BALF, bronchoalveolar lavage fluid.

PA revealed metabolic pathways consistently disrupted across both human and animal models, including: 1) alanine, aspartate and glutamate metabolism; 2) arginine biosynthesis; 3) glycine, serine and threonine metabolism; 4) glutathione metabolism; 5) glyoxylate and dicarboxylate metabolism; and 6) one-carbon pool by folate metabolism (Table 3).

##### 3.3.4.2 Metabolite variations

Despite the overlap in significant pathways detected, the direction of metabolite changes within these shared pathways was not always conserved across studies. Supplementary Tables S9A–V provides a detailed summary of the directional changes and study-specific characteristics for each metabolite identified within the significant pathways across all research models and sample matrices.

## 4 Discussion

The comparison of differential metabolites between human and animal model studies revealed relatively low overlap percentages (collectively 4.7%). However, these results should be interpreted with caution. The observed overlap is inherently dependent on the metabolites captured by individual studies, which are shaped by factors including experimental design, choice of analytical platform, sample matrices analysed, and biological variability. Importantly, a considerably larger number of metabolomic studies have been conducted in humans compared to animal models, resulting in an asymmetry of data availability. Consequently, the limited overlap and small coverage observed for animal models likely reflect not a lack of biological relevance or translational potential, but rather the current paucity and heterogeneity of animal metabolomic studies.

The following sections explore some of these variables in more detail to clarify their influence on study outcomes and the interpretation of cross-species metabolomic data.

### 4.1 Species-specific observations

*Mtb* infection is known to induce metabolic alterations in its host (Ding et al., 2020). Host-directed changes, including altered glycolysis and amino acid metabolism, support antimicrobial responses, cytokine production, and immune cell activation (Kumar et al., 2019; Shi et al., 2016). In contrast, *Mtb* can manipulate lipid and amino acid pathways to evade immune clearance and create a niche for persistence (Borah Slater et al., 2023; Kumar et al., 2019; Shi et al., 2016).

Comparative analyses across species suggest some conserved metabolic responses to *Mtb* infection. From PA, reduced circulating amino acid levels were consistently observed across research models, particularly in alanine, aspartate, and glutamate metabolism, as well as glycine, serine, and threonine metabolism (Table 3). These pathways are central to *Mtb* pathogenesis: alanine, aspartate, and glutamate metabolism serve as essential carbon and nitrogen sources for *Mtb* amino acid biosynthesis, whereas disruption of glycine, serine, and threonine metabolism, together with cysteine and methionine metabolism, may reflect host strategies to restrict nutrient availability (Borah Slater et al., 2023).

Furthermore, PA also highlighted consistent disruptions across all research models in the one-carbon pool by folate metabolism, underpinned by altered serine, glycine, and cysteine pathways (Table 3). Such disruptions may both restrict nutrients essential for *Mtb* growth and enhance pro-inflammatory immune responses (Borah Slater et al., 2023). In addition, guinea pigs and mice exhibited similar disrupted arginine biosynthesis and glutathione metabolism compared to human studies (Table 3). Both of these pathways are important for host defence against *Mtb*-induced oxidative stress (McKell et al., 2021; Young et al., 2018).

Tissue-specific alterations were also evident in mice, with disruptions in pyruvate metabolism, tricarboxylic acid (TCA) cycle pathways, and valine, leucine and isoleucine degradation pathways (Table 3). During TB infection, immune cells undergo a Warburg-like shift from oxidative phosphorylation to glycolysis to sustain activation and survival (Chaudhary et al., 2024; Shin et al., 2011). This shift disrupts pyruvate metabolism and remodels the TCA cycle, characterised by succinate accumulation, which drives pro-inflammatory signalling (Yu et al., 2023), alongside changes in citrate and malate (Chaudhary et al., 2024; Shin et al., 2011). Branched-chain amino acid metabolism is also affected, reflecting both *Mtb*’s use of isoleucine, leucine, and valine for growth and the host’s attempt to limit nutrient access (Chaudhary et al., 2024).

In contrast, cattle (included in only one study) showed fewer disrupted pathways, though glyoxylate and dicarboxylate metabolism [linked to altered energy metabolism (Rahman and Schellhorn, 2023)] was similarly affected as observed in both human and mouse studies (Table 3).

Among the animal models, mice appear to have the highest degree of disrupted pathway overlap with humans. This aligns with the distinct metabolite profiles, where mouse lung tissue captured the largest subset of overlapping TB-associated differential metabolites (3.8%), followed by guinea pig serum and lung tissue, both with 0.5% overlap (Supplementary Table S7).

**FIGURE 6:**
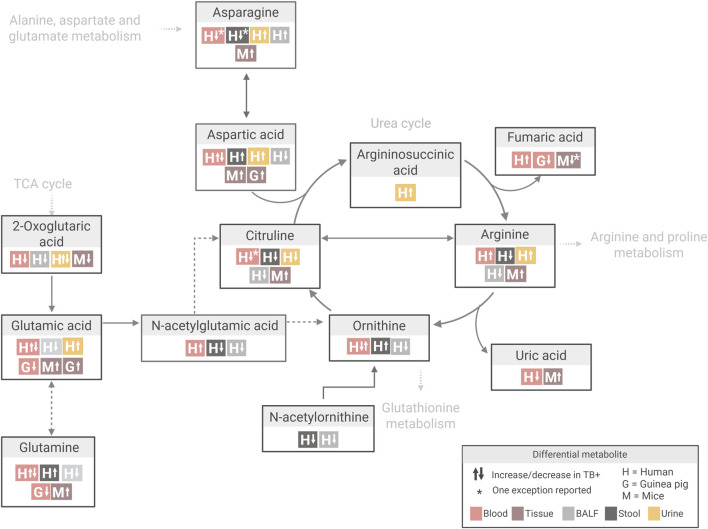
Overview of differential metabolite changes in arginine biosynthesis during *Mtb* infection across human and animal models. Created in Biorender, https://BioRender.com/bj25qqj.

Further examination of metabolite-specific variation within these pathways highlighted important interspecies differences. For instance, glutamine levels (a key precursor in arginine biosynthesis) were mostly decreased in *Mtb*-infected guinea pigs (aligned with observations in human patients) but increased in mice (Figure 6; Supplementary Table S9A). This divergence may reflect species-specific differences in arginine precursor pathways, where humans rely more on glutamine and proline, while mice favour arginine and ornithine (Marini, 2012; Morris, 2016).

### 4.2 Variances based on different group comparisons

In animal model studies, group comparisons are commonly made between *Mtb*-infected animals and HC, which is fundamental for understanding TB processes (Li and Li, 2023; Omenn et al., 2012; Singh and Gupta, 2018). This study design is preferred because it isolates the effects of *Mtb* infection by using HC as a baseline, allowing for more confident attribution of observed changes—such as shifts in metabolism (Weiner 3rd et al., 2012), immune responses, or tissue pathology (Li and Li, 2023; Omenn et al., 2012; Singh and Gupta, 2018) — directly to the infection itself. This approach helps to minimise confounding variables (Fernández-García et al., 2020) and ensures that experimental differences are due to the infection itself rather than unrelated physiological or environmental factors.

In contrast, human studies often incorporate group comparisons between TB patients and either HC or non-TB individuals. This approach not only enables investigation into disease mechanisms but also facilitates the development of clinically relevant biomarkers for differentiating TB from other conditions or diseases presenting with overlapping symptoms (Weiner 3rd et al., 2012).

Interestingly, a few metabolites—such as adenine, asparagine, lactic acid, leucine, methionine, phenylalanine, phosphocholines—showed similar directional changes in both animal model TB-infected lung tissue (Fernández-García et al., 2020; Shin et al., 2011; Somashekar et al., 2011; Somashekar et al., 2012; Zhang et al., 2024b) and BALF (Li et al., 2024), compared to HC (Supplementary Tables S9A,E,H,L,N,P,Q,S,V).

However, discrepancies were observed between these matrices for other metabolites, including alanine, aspartic acid, citrulline, creatine, glutamine, pyroglutamic acid, succinic acid, tyrosine, uracil, and xanthine (Fernández-García et al., 2020; Li et al., 2024; Shin et al., 2011; Somashekar et al., 2011; Somashekar et al., 2012; Zhang et al., 2024b) (Supplementary Tables S9A,B,C,D,G,I,J,K,M,N,O,P,Q,R,U). Interestingly, several differential BALF metabolites—including betaine, choline, cysteine, glyceric acid, methionine, proline, and sphingosine—showed opposite trends for the different comparison groups used (TB vs. HC controls and TB vs. non-TB) (Li et al., 2024) (Supplementary Tables S9B,E,G,H,I,J,N,O,T).

From these observations, it is notable that contrasting trends were frequently observed between comparisons to different control groups, namely HC and non-TB. In this review, the term non-TB was broadly defined to include symptomatic TB suspects who tested negative, individuals with other diseases, and cases of non-tuberculous mycobacterial (NTM) infections (Table 1). Li et al. (2024) explored the interplay between lung microbial communities and infections by *Mtb* and NTM. They deduced that NTM infections induce distinct shifts in the lung microbiota and disrupt metabolism to support a niche environment for persistent NTM infection, distinct from that observed during *Mtb* infection.

**FIGURE 7:**
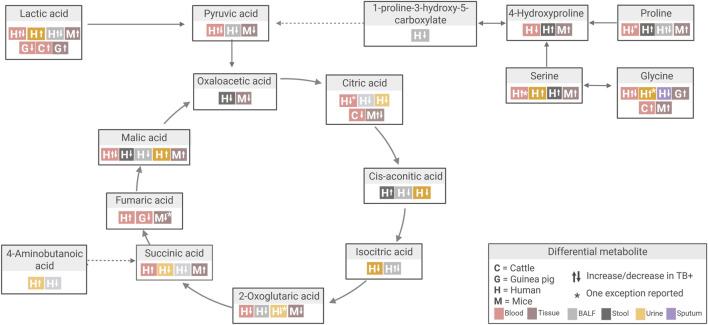
Overview of differential metabolite changes in Tricarboxylic acid (TCA) cycle metabolism during *Mtb* infection across human and animal model studies. Created in Biorender, https://BioRender.com/k14ouej.

Serine is another notable example. This metabolite showed consistent increases across nearly all studies comparing TB to control groups (Conde et al., 2022; Deng et al., 2021; Fernández-García et al., 2020; Rai et al., 2023; Sa et al., 2024; Vrieling et al., 2019; Yu et al., 2024; Zhang et al., 2024a) (Figure 7; Supplementary Table S9E). However, one study reported decreased plasma serine levels in TB vs. non-TB controls (Sun et al., 2016). This study focused on paediatric TB (children under 14 years), while others included adults (aged 17–69; Zhang et al. (2024a) did not specify age). Given the diagnostic challenges in children and emerging evidence suggesting age-related differences in TB biomarkers, this discrepancy may be related to such age-related variations (Sun et al., 2016).

Altogether, these findings highlight the complexity and importance of control group selection in comparative metabolomics. Non-TB populations are inherently heterogeneous and may harbour undiagnosed infections or other metabolic disturbances that confound interpretation.

### 4.3 Variation due to induced Mtb infection protocols

Another important consideration when using animal models in TB metabolomics studies is the infection characteristics of *Mtb* itself, as these factors could contribute to variation in the TB-induced metabolome observed.

In the reviewed studies, some conserved metabolite responses were observed despite differences in animal species, species strains, and *Mtb* infection strains. For instance, aspartic acid levels were consistently increased in two independent studies involving C57BL/6 mice infected with *Mtb* H37Rv, compared to controls (Fernández-García et al., 2020; Zhang et al., 2024b) (Supplementary Table S9A). Similarly, metabolites such as glycine and glutathione showed similar changes in both mice and guinea pigs, across different species strains infected with either *Mtb* H37Rv and W-Beijing strains (Fernández-García et al., 2020; Shin et al., 2011; Somashekar et al., 2011; Somashekar et al., 2012) (Supplementary Tables S9G, I). Furthermore, glutamic acid, glutamine, succinic acid, and oxaloacetic acid exhibited similar directional variation in liver and lung tissues from mice and guinea pigs infected with *Mtb* H37Rv (Supplementary Table S9A) (Fernández-García et al., 2020; Shin et al., 2011; Somashekar et al., 2011; Somashekar et al., 2012; Zhang et al., 2024b).

However, the infectious strains used in these studies represent only two of the eight recognised *Mtb* lineages: H37Rv and Erdman, both belong to Lineage 4 (Euro-American), while W-Beijing represents Lineage 2 (East Asian) (Luo et al., 2015). This raises concerns about the broader representativeness of these strains, particularly the widely used H37Rv strain, in reflecting the species diversity observed in human TB. To enhance translational relevance, it has been recommended that future animal model studies incorporate a more diverse set of *Mtb* strains (Chiner-Oms et al., 2018; O'Toole and Gautam, 2017).

The route of infection is also recognised as a factor that can significantly affect disease pathology and immune responses (Flynn, 2006). To date, however, no studies have specifically explored the metabolic responses associated with different infection routes in TB metabolomics. Furthermore, due to the considerable variability in experimental designs among the nine animal model studies included in this review, the impact of the infection route could not be meaningfully evaluated across studies.

Beyond infection strain and route, both infection duration and dose are additional variables that can affect the metabolic responses observed in TB. Some discrepancies in the differential metabolites detected in animal models could reflect different stages of disease progression. Fumaric acid, a key intermediate in the TCA cycle, showed temporal variation in both mouse and guinea pig models (Fernández-García et al., 2020; Shin et al., 2011; Somashekar et al., 2012) (Figure 7; Supplementary Table S9U). In one mouse study, fumaric acid was decreased at 4 weeks post-infection (TB_W4), attributed to succinate dehydrogenase and malate dehydrogenase inhibition. This coincided with the accumulation of succinic acid and malic acid, reflecting a pro-inflammatory metabolic profile (Fernández-García et al., 2020). Notably, between four and 9 weeks post-infection (TB_W4 to TB_W9), fumaric acid levels increased while succinic acid and malic acid levels decreased, a pattern interpreted as either inflammation resolution or a metabolic shift associated with chronic infection (Fernández-García et al., 2020). Coinciding with the four-week time point, similar decreases in fumaric acid were observed in mouse lung and spleen tissue (Shin et al., 2011) and in guinea pig serum (Somashekar et al., 2012) at approximately 4 weeks post-infection.

### 4.4 Metabolomics workflow variables

In addition to species differences, the choice of sample matrix had a marked impact on the metabolic signatures observed in TB. Compared to human studies, the reviewed animal model studies showed limited diversity in sample matrices. However, animal models offered the advantage of using tissue samples, particularly from the lung, enabling direct investigation of organ-specific pathology and TB-associated metabolic alterations.

Certain pathways appeared to be exclusively altered in specific sample matrices. For example, cysteine and methionine metabolism were significantly altered in urine, while pyrimidine metabolism showed significant changes in BALF. Linoleic acid and sphingolipid metabolism were significantly altered in blood (Table 3). Additionally, branched-chain amino acid metabolism (Valine, leucine and isoleucine degradation) and phenylalanine, tyrosine and tryptophan biosynthesis were indicated to significantly alter only in mice liver and spleen tissues (Table 3).

Variations in metabolites and the directionality of metabolic alterations across different matrices (blood, sputum, urine, and stool) reflect the fact that the metabolic response to *Mtb* infection involves both localised and systemic effects. Local changes, such as those observed in sputum, reflect lung-specific alterations, while systemic responses are captured in blood, urine, and stool, indicating broader physiological effects.

Interestingly, metabolites from mouse lung tissue often aligned with findings from human BALF, particularly within pathways such as alanine, aspartate and glutamate metabolism; arginine and proline metabolism; glutathione metabolism; and glycine, serine and threonine metabolism (Table 3; Supplementary Table S9). This suggests that, despite differences in sample matrices, meaningful comparative insights can still be drawn when matrices reflect related biological compartments or processes. Such cross-matrix comparisons may be particularly valuable in bridging findings between animal models and human studies.

Beyond matrix selection, the specific metabolomics approach and analytical platforms used also significantly influence which metabolites are detected in animal models. In hypothesis-generating research, untargeted, multi-platform approaches are particularly valuable when broad metabolite coverage is desired. For example, Fernández-García et al. (2020) employed an untargeted, multi-platform approach combining CE-MS, GC-MS, and LC-MS, which enabled high metabolite coverage in mouse lung tissue and facilitated the discovery of novel disease mechanisms (Supplementary Table S5). Conversely, for research targeting specific metabolite classes or pathways, targeted or semi-targeted approaches may be more appropriate. This was demonstrated in the mouse model study by Jain et al. (2007), where a semi-targeted approach was employed to extract and analyse lipids in the context of lipid metabolism.

### 4.5 Animal model selection: advantages, limitations, and future prospects

Several factors contribute to the limited use of animal models in metabolomics studies aimed at TB characterisation. A major challenge lies in the translational relevance of many animal models, as the pathological manifestations of TB in most models do not fully reflect the complexity observed in human disease.

In addition to translational constraints, logistical and practical constraints also likely discourage animal-based metabolomics studies. In contrast to human studies, which can leverage existing biobanks or clinic-derived samples, animal studies require time-consuming and costly infection protocols and husbandry prior to sample collection. These requirements increase costs and reduce feasibility for large-scale or longitudinal metabolomic profiling. Moreover, metabolic processes are inherently sensitive to environmental influences. While controlled conditions in animal facilities help reduce variability, they may inadvertently omit key host-environment interactions that contribute to disease manifestation in human populations. Thus, controlled environments represent both a strength and a limitation, depending on the specific research question.

Despite these challenges, the choice of animal model remains a critical determinant of metabolomic results. Different species capture distinct aspects of TB pathology and host–pathogen interactions, and their careful selection is essential to maximise both biological insight and translational relevance.

Mouse models are widely used in TB metabolomics to investigate disease characteristics, drug mechanisms, and potential toxicities (Du Preez et al., 2019). They offer major practical advantages, such as cost-effectiveness, ease of handling, and availability of inbred strains (Corleis et al., 2023). However, a key limitation is their inability to fully replicate human TB pathology, particularly the absence of caseating granulomas and cavitary lesions, which are characteristic of human TB (Gong et al., 2020; Singh and Gupta, 2018).

Guinea pigs are more susceptible to *Mtb* than many other animal models, requiring only a small bacterial inoculum to establish infection (Clark et al., 2014; Li and Li, 2023). Upon infection, they develop granulomas with central necrosis surrounded by lymphocytes, macrophages, and multinucleated giant cells enclosed by a fibrotic capsule—closely resembling human TB pathology. This histopathological similarity makes guinea pigs valuable for evaluating TB pathogenicity, as well as for testing candidate treatments and vaccines (Dharmadhikari and Nardell, 2008). For instance, Palanisamy et al. (2008) assessed the virulence of different *Mtb* strains in guinea pigs by comparing survival time, bacterial loads in organs like the lungs, spleen, and lymph nodes, and the severity of pulmonary and extrapulmonary lesions. Guinea pigs do, however, lack many human-specific immune reagents important for investigating underlying TB mechanisms and they do not manifest the full clinical spectrum, including LTBI (Li and Li, 2023; Zhan et al., 2017).

Cattle also represent a highly translational model, as *M. bovis* infection closely mimics *Mtb* infection in humans. Both species develop granulomatous lesions featuring caseous necrosis, mineralisation, and fibrosis, predominantly in the lungs and regional lymph nodes (Pollock et al., 2006). The bovine immune response is Th1-dominant, with CD4^+^ and CD8^+^ T-cells promoting IFN-γ production for bacterial control, while γδ T-cells play a role in early containment of infection. Lesion progression in cattle follows a timeline that is comparable to human disease following *Mtb* infection (Pollock et al., 2006). Cattle, therefore, serve as a valuable translational bridge between small animal models and human clinical studies, particularly in the context of vaccine development and host-pathogen interaction research (Pollock et al., 2006). Moreover, the availability of well-characterised immunological reagents for bovine TB supports detailed, reproducible investigations. Cattle also enable the collection of large-volume blood samples, which is particularly advantageous for metabolomics. However, their use in large-scale TB metabolomics research remains limited due to the considerable logistical demands, housing requirements and high associated costs (Li and Li, 2023).

The current limited use of animal models in metabolomics does, however, restrict the ability to identify the most suitable animal model and optimise experimental designs for TB metabolomics. Further investigation is therefore required, including exploring alternative animal models that have demonstrated value in TB pathogenesis research, such as the New Zealand rabbit, Cynomolgus macaque or Rhesus monkey, Chinese tree shrew, and Wistar rat (Zhan et al., 2014; Zhan et al., 2017). At the same time, systematic evaluation of variables such as infection route, strain, dose, and duration will be critical for determining how experimental conditions shape metabolomic profiles and their translational relevance to human TB.

Beyond the selection and optimisation of animal models, the choice of sample matrix and metabolomics approach also plays a pivotal role in shaping the insights gained and can further enhance the translational value of these models. In line with established practices in hypothesis-generating research, untargeted metabolomics strategies covering diverse metabolite classes are especially valuable for uncovering unknown or novel disease mechanisms. Greater metabolome coverage can be achieved using multi-platform metabolomics approaches and by incorporating diverse sample matrices, enabling the investigation of both systemic and local metabolic changes.

Furthermore, multi-omics approaches that integrate metabolomics, transcriptomics, and proteomics are emerging as powerful tools to deepen understanding of TB pathogenesis. For example, Duffy et al. (2019) demonstrated the value of combining metabolomics and transcriptomics datasets from HHCs across multiple African sites, some of whom developed TB while others remained TB-negative. This integrated approach provided complementary insights into TB progression. Incorporating immunometabolic pathways, the researchers developed biologically interpretable multi-omics signatures that outperformed existing models in predicting TB-related pathology and bacterial load in rhesus macaque vaccine challenge studies. Applying similarly comprehensive datasets from animal models infected with *Mtb* could reveal correlative insights that enrich current knowledge of host-pathogen interactions and disease mechanisms.

## 5 Conclusion

Collectively, these findings suggest that animal models can, to some extent, recapitulate key metabolic features of human TB, although outcomes depend heavily on species, strain, sample type, and the chosen metabolomics approach. Despite their underutilisation, these models hold considerable potential for metabolomics-based TB disease characterisation. This is supported by insights from the limited number of TB-focused studies reviewed here, as well as from broader metabolomics research applying animal models to other human diseases. Altogether, this systematic review provides a comprehensive overview of the current use of animal models in TB metabolomics for disease characterisation and highlights key considerations for advancing these models toward translational relevance.
