# Supplementary material for: Animal models in tuberculosis metabolomics: a systematic review of current evidence and the road to translational relevance
Source: Front Mol Biosci. 2025 Oct 15;12:1688882. doi: 10.3389/fmolb.2025.1688882 (PMC12568366; doi:10.3389/fmolb.2025.1688882)
Supplement: Supplementary file 1 [file Supplementaryfile1.docx]

**Animal models in tuberculosis metabolomics: A systematic review of current evidence and translational relevance**

Rochelle Caudron^1^, Ilse du Preez^2^, Laneke Luies^1^ and Monique Opperman^2^*

^1^Biomedical and Molecular Metabolism Research, Faculty of Natural and Agricultural Sciences, North-West University, South Africa.

^2^Centre for Human Metabolomics, Desmond Tutu School of Medicine, Faculty of Health Science, North-West University, South Africa.

*Correspondence:

Monique Opperman ([25902806@mynwu.ac.za](mailto:25902806@mynwu.ac.za))

Short title: Animal models in TB metabolomics: Systematic review and translational insights

1. **Methods**
   1. **Search strings used for each specific database**

***PubMed*:** (("Mycobacterium tuberculosis"[Mesh] OR "Mycobacterium Infections"[Mesh] OR "Tuberculosis"[Mesh] OR "Latent Tuberculosis"[Mesh] OR "Tuberculosis, Pulmonary"[Mesh]) AND ("Metabolomics"[Mesh] OR "Metabolome"[Mesh]))

***Scopus*:** TITLE-ABS (("mycobacterium tuberculosis" OR "Mycobacterium tuberculosis" OR "Mycobacterium Infections" OR "Tuberculosis" OR "Latent Tuberculosis" OR "Tuberculosis, Pulmonary") AND ("Metabolomics" OR "Metabolome"))

***Web of Science*:** (AB=(mycobacterium tuberculosis OR "Mycobacterium tuberculosis" OR "Mycobacterium Infections" OR "Tuberculosis" OR "Latent Tuberculosis" OR "Tuberculosis, Pulmonary") AND AB=("Metabolomics" OR "Metabolome")
